# CD4 T-Cell Responses in Primary HIV Infection: Interrelationship with Immune Activation and Virus Burden

**DOI:** 10.3389/fimmu.2016.00395

**Published:** 2016-09-29

**Authors:** Mathieu F. Chevalier, Céline Didier, Pierre-Marie Girard, Maria E. Manea, Pauline Campa, Françoise Barré-Sinoussi, Daniel Scott-Algara, Laurence Weiss

**Affiliations:** ^1^Institut Pasteur, Régulation des Infections Rétrovirales, Paris, France; ^2^Université Paris Diderot, Sorbonne Paris Cité, Paris, France; ^3^AP-HP, Hôpital Saint Antoine, Paris, France; ^4^AP-HP, Hôpital Européen Georges Pompidou, Paris, France; ^5^Université Paris Descartes, Sorbonne Paris Cité, Paris, France

**Keywords:** HIV-specific CD4 T cells, acute HIV infection, viral load, generalized immune activation, antiretroviral therapy, early ART initiation

## Abstract

Early events during primary HIV infection (PHI) are thought to influence disease outcome. Although a growing body of evidence suggests a beneficial role of HIV-specific CD4 help in HIV infection, it is unclear how early viral replication, systemic immune activation, and antiretroviral therapy (ART) may shape CD4 T-cell responses during PHI, and whether HIV-specific CD4 responses contribute to the high immune activation observed in PHI. Twenty-seven patients with early PHI were included in a prospective longitudinal study and 12 of them received ART after enrollment. Fresh peripheral blood mononuclear cells were used for measurement of *ex vivo* T-cell activation and of cytokine-producing CD4 T-cells following stimulation with PMA/ionomycin or HIV-1-gag-p24 antigen. Patients were segregated based on CD8 T-cell activation level (i.e., % HLA-DR^+^CD38^+^ CD8 T-cells) at baseline (BL). Patients with lower immune activation exhibited higher frequency of bulk CD4 T-cells producing IFN-γ or IL-17 and higher effector-to-regulatory cell ratios. No differences were found in HIV-specific CD4 T-cell frequencies. In contrast, segregation of patients based on plasma viral load (pVL) revealed that patients with higher pVL showed higher cytokine-producing HIV-specific CD4 responses. Of note, the frequency of IFN-γ^+^ HIV-specific CD4 T cells significantly diminished between BL and month 6 only in ART-treated patients. However, early treatment initiation was associated with better maintenance of HIV-specific IFN-γ^+^ CD4 T-cells. These data suggest that HIV-specific CD4 responses do not fuel systemic T-cell activation and are driven by viral replication but not able to contribute to its control in the early phase of infection. Moreover, our data also suggest a benefit of early treatment for the maintenance of HIV-specific CD4 T-cell help.

## Introduction

CD4 T helper cells are crucial to orchestrate immune responses to viral infections by recruiting key immune cells into tissues, by providing help for expansion and function of B cells and CD8 T cells, and by direct cell-mediated cytotoxicity (1). While extensive research deciphered the ability of CD8 T cells to control HIV viral replication, CD4 T cells have long been neglected as effectors in HIV immunity owing to the fact that they are the major targets of the virus (2). Nevertheless, several studies ascribed a protective role to HIV-specific CD4 T cells with regard to viremia and disease progression (3–7), as well as in vaccine-mediated protection against HIV acquisition (8, 9). T helper responses during primary HIV infection (PHI) may be of particular interest as early events are thought to influence disease outcome. Although HIV-specific proliferative CD4 T-cell responses are quite limited in most patients during PHI (10), cytokine-producing virus-specific CD4 T cells have been clearly identified during PHI in several recent studies (4, 11–13). Of note, HIV-specific CD4 and CD8 T cells arise with distinct kinetics during PHI, with specific CD4 T cells reaching their maximal frequency within few weeks post-infection, while CD8 T cells gradually increase for several months (11).

Although it was first considered “unlikely” that early established CD4 T cell responses were related to the control of viremia (13), three recent studies clearly reported a beneficial role for HIV-specific CD4 T cells in PHI with regard to viral set point (4) and clinical progression (6, 12), in line with interventional experiments conducted in acute SIV infection (14).

Thus, a growing body of evidence supports a highly important role for HIV-specific CD4 T cells, which alleviates the fear that activation of these cells may fuel viral spreading and accelerate disease progression, as suggested (15). Conversely, it remains unclear how viral replication and systemic immune activation – both particularly high during PHI – may shape effector CD4 T-cell responses, and whether HIV-specific CD4 responses contribute to the immune activation in early infection. In this study, we thus investigated both polyclonal and HIV-specific cytokine-producing CD4 T cells as a function of the immuno-virological status of patients diagnosed with early acute HIV infection. We describe here interrelationships between viral replication, immune activation, and functional CD4 T cell responses in early infection.

## Materials and Methods

### Study Population

Individuals with acute HIV infection were recruited in a multicenter prospective study. These patients have been described elsewhere (16–18). Acute HIV infection was defined by <3 bands on HIV Western Blot, a positive p24 antigenaemia and/or a detectable plasma HIV-RNA with a negative or weakly positive ELISA. The estimated date of infection was calculated as 2 weeks before onset of symptoms for patients with symptomatic PHI or 4 weeks before the first positive Western Blot. At enrollment [baseline (BL)], all patients were treatment-naive. Some of the patients initiated antiretroviral treatment (ART) within 3 months, based on CD4 cell counts and the decision of both physicians and patients. Peripheral blood was collected in EDTA-containing tubes at BL and at month 6 of follow-up (M6). Written informed consent was provided by all patients, and the study was approved by the ethical committee of Ile de France II. Plasma HIV-RNA levels and CD4 counts were determined in the four recruiting hospitals, using the locally available technique with a maximum detection limit of 20 copies/mL.

### Flow Cytometric Analysis

Fresh peripheral blood mononuclear cells (PBMCs) were purified by density gradient centrifugation (Isopaque-Ficoll) within 2–4 h after blood sampling. Only freshly isolated cells were used in this study to avoid any bias due to cryopreservation. Cells were stained using multicolor panels and analyzed by flow-cytometry (LSRII cytometer, Becton Dickinson) as described previously (19). The following monoclonal antibodies (mAbs) conjugated to PE Texas Red (ECD), allophycocyanin (APC)–Hilite7 (H7), Alexa Fluor 488 (AF488), Alexa Fluor 700 (AF700), APC, peridinin chlorophyll protein–cyanin 5.5 (PerCP–Cy5.5), fluorescein isothiocyanate (FITC), Alexa Fluor 647 (AF647), and phycoerythrin–cyanin 7 (PE–Cy7) were used at predetermined optimal concentrations: anti-CD3–ECD (Beckman Coulter); anti-CD4–APC–H7, anti-CD8–AF488, anti-CD8–AF700, anti-CD25–APC, anti-CD38–APC, anti-HLA-DR–PerCP–Cy5.5, anti-IFN-γ–AF700, anti-IL-2–FITC, anti-IL-17–AF647 (BD Biosciences); anti-CD127–PE–Cy7, anti-FoxP3–AF700 (eBiosciences); and anti-Ki-67–FITC (Dako).

FcR Blocking Reagent (Miltenyi Biotec) was used to block unwanted binding of antibodies and increase the staining specificity of cell surface antigens. For intracellular staining of IFN-γ, IL-2, IL-17, FoxP3, or Ki-67, cells were fixed and permeabilized using the “FoxP3 Staining Buffer Set” (eBioscience) according to the manufacturer’s recommendations. Analyses were performed using the FlowJo software (TreeStar).

### Measurement of CD4 T-Cell Responses

CD4 T-cell isolation from fresh blood samples was performed prior to density gradient centrifugation by incubating the blood with the “RosetteSep human CD4 T-cell enrichment” antibody cocktail (Stem Cell Technologies) according to the manufacturer’s recommendations. For assessment of “polyclonal” responses, freshly isolated CD4 T cells were stimulated with PMA (5 ng/mL) and ionomycin (1 μg/mL) at 37°C for 5 h. After 2 h of culture, brefeldin A (5 μg/mL) (Sigma-Aldrich) was added. Measurement of HIV-specific CD4 T-cell responses was performed by stimulating the cells with recombinant HIV-1 gag p24 protein (5 μg/mL, provided by the *Agence Nationale de la Recherche sur le Sida*) in the presence of autologous monocytes (monocyte/CD4 T cell ratio of 1:10) for 3 days. Intracellular cytokine staining (ICS) was performed as described above.

### Statistical Analysis

Non-parametric tests were used to avoid the impact of potential outlier values in a small study. Comparisons between groups of patients were performed using the Mann–Whitney test. The Wilcoxon matched pairs test was used to estimate the changes in the different variables throughout the follow-up (BL vs. month 6). *P*-values below 0.05 were considered statistically significant.

## Results

### Study Population

Twenty-seven patients with early untreated PHI were included in a prospective longitudinal study and followed up for 6 months, as previously described (16, 18). Median (IQR) of estimated time post-infection at BL was 42 days (29–50). Twelve patients received ART after enrollment. Patients’ clinical characteristics at BL and at month 6 (M6) of follow-up are shown in Table 1.

**Table 1:** **Patients’ characteristics**.

Time points	Patients	HIV-1 RNA (log_10_ copies/mL)	CD4 count (cells/mm^3^)
Baseline	Untreated	5.65 (4.57–6.25)	490 (337–615)
	*n* = 27		
Month 6	Untreated	4.40 (3.50–4.80)	669 (457–725)
	*n* = 13		
Month 6	ART-treated	1.30 (1.00–1.33)	750 (593–783)
	*n* = 12		

*Data are expressed as median (IQR)*.

*Two patients were lost to follow-up*.

### Patients with a Higher CD8 T-Cell Activation Level Exhibit Lower Polyclonal CD4 T-Cell Responses

**Figure 1:**
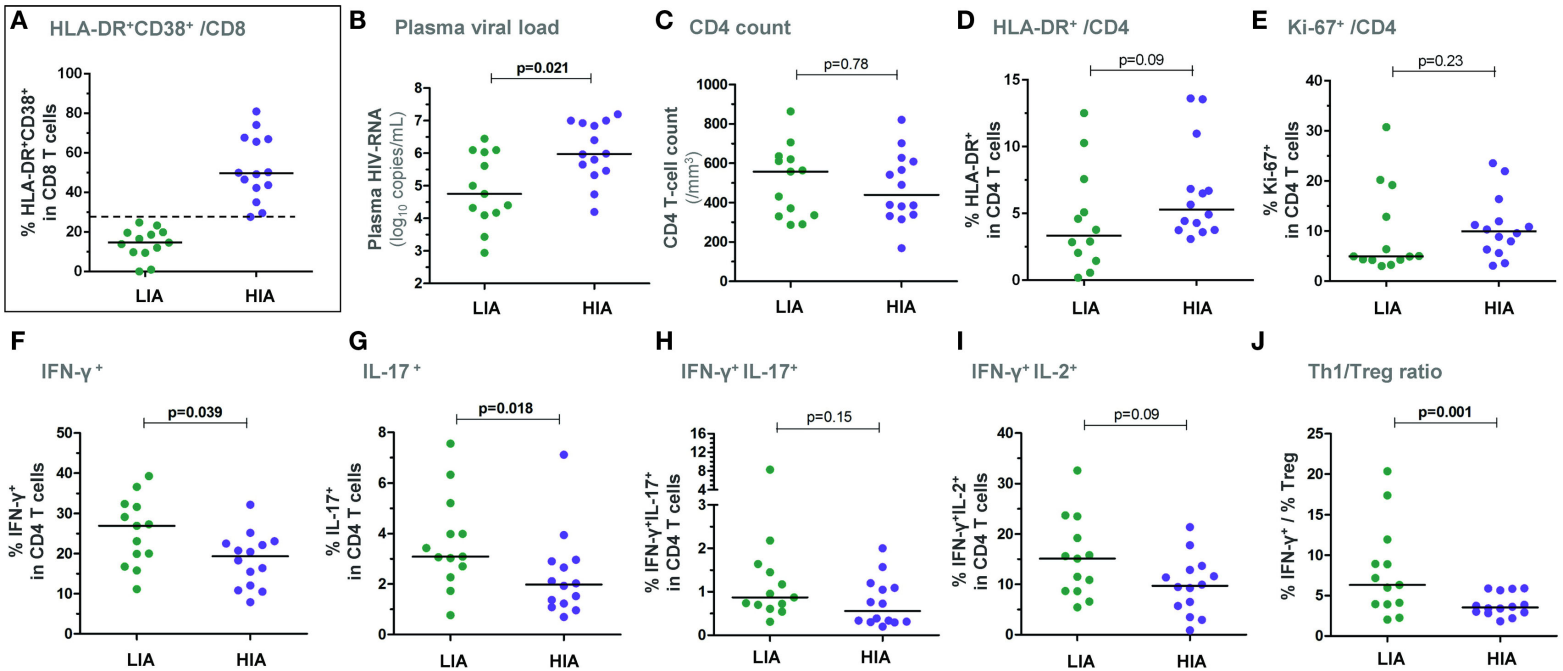
**Patients with higher levels of CD8 T-cell activation show lower *ex vivo* CD4 T-cell responsiveness in early PHI**. Patients were segregated based on the level of *ex vivo* CD8 T-cell activation at baseline: the group with higher immune activation (HIA) was defined as % HLA-DR^+^CD38^+^ CD8 T cells ≥ median and the group with lower immune activation (LIA) as % HLA-DR^+^CD38^+^ CD8 T cells < median **(A)**. Plasma viral load **(B)**, CD4 T-cell counts **(C)**, % HLA-DR^+^ CD4 T cells **(D)**, and % Ki-67^+^ CD4 T cells **(E)** are depicted in both patient groups. Freshly isolated CD4 T cells (at baseline) were stimulated with PMA/ionomycin for 5 h and frequencies of IFN-γ^+^ **(F)**, IL-17^+^ **(G)**, IFN-γ^+^IL-17^+^ **(H)**, as well as of IFN-γ^+^IL-2^+^ **(I)** CD4 T cells are shown in HIA and LIA patients. The ratios between effector Th1 responses (% IFN-γ^+^) and *ex vivo* CD25^+^CD127^low^FoxP3^+^ regulatory T cells are also depicted **(J)**. *P*-values of Mann–Whitney tests are indicated on each panel.

Two groups of patients were segregated based on the level of CD8 T-cell activation at BL: (i) a group with a high immune activation (“HIA patients,” *n* = 14), i.e., % HLA-DR^+^CD38^+^ CD8 T cells ≥ median (27.6%) and (ii) a group with a low immune activation (“LIA patients,” *n* = 13), i.e., % HLA-DR^+^CD38^+^ among CD8 T cells < median (Figure 1A). As expected, HIA patients had also a higher frequency of CD8 T cells expressing the activation/proliferation marker Ki-67 as compared with LIA patients (median 58.3% vs. 20.1%; *p* = 0.0006, data not shown). Also as expected, HIA patients had higher plasma HIV-RNA levels (*p* = 0.021, Figure 1B); in contrast, CD4 T-cell counts were similar in both groups (Figure 1C). There was only a trend to a higher proportion of HLA-DR-expressing CD4 T cells in HIA patients and no significant difference for Ki-67^+^ CD4 T cells (Figures 1D,E). The estimated time from infection did not differ between groups (data not shown).

Following 5 h PMA/ionomycin activation of freshly isolated CD4 T cells (Figure S1A in Supplementary Material), HIA patients exhibited a significantly lower frequency of IFN-γ^+^ and IL-17^+^ single positive CD4 T cells as compared to LIA patients (*p* = 0.039 and *p* = 0.018, respectively), while frequencies of IFN-γ^+^IL-17^+^ double positive cells were similar (Figures 1F–H). Although there was a trend to a lower frequency of IFN-γ^+^IL-2^+^ double positive CD4 T cells in HIA patients (*p* = 0.09, Figure 1I), IL-2 expression did not differ between groups (data not shown).

Importantly, frequencies of HIV-specific CD4 T cells − as measured by IFN-γ, IL-2, or IL-17 expression following gag-p24 stimulation – were similar in HIA and LIA patients (data not shown).

As natural regulatory T cells (Tregs) can dampen CD4 T-cell responses, it was of interest to assess the balance between effector CD4 T cells and Tregs. We thus measured the *ex vivo* frequency of Treg cells, defined as CD25^+^CD127^low^FoxP3^+^ CD4 T cells. Interestingly, HIA patients exhibited lower effector/regulatory T-cell balance when considering the Th1/Treg ratio (Figure 1J) as well as the Th17/Treg ratio [data not shown and Ref. (16)].

### Patients with a Higher Plasma Viral Load Exhibit Higher HIV-Specific CD4 T-Cell Responses

**Figure 2:**
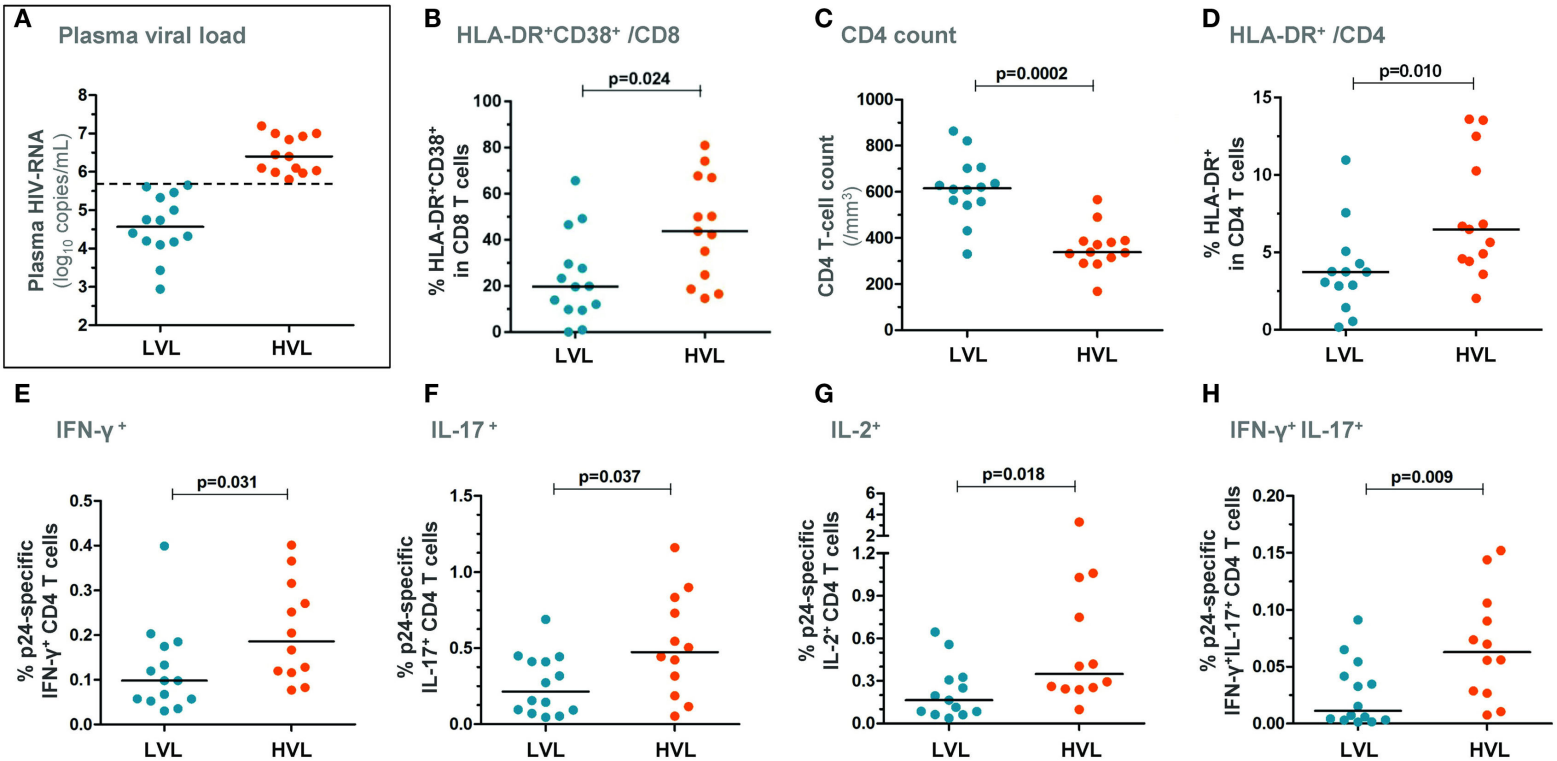
**Patients with higher plasma viral load show higher HIV-specific CD4 T-cell responses in early PHI**. Patients were segregated based on the level of plasma viral load at baseline: the group with higher viral load (HVL) was defined as blood HIV-RNA copies/mL > median and the group with lower viral load (LVL) as HIV-RNA copies/mL ≤ median **(A)**. CD8 T-cell activation levels (% HLA-DR^+^CD38^+^ CD8 T cells) **(B)**, CD4 T-cell counts **(C)**, and % HLA-DR^+^ CD4 T cells **(D)** are depicted in both patient groups. Freshly isolated CD4 T cells (at baseline) were stimulated with HIV-1 gag p24 protein for 72 h and frequencies of IFN-γ^+^ **(E)**, IL-17^+^ **(F)**, IL-2^+^ **(G)**, as well as of IFN-γ^+^IL-17^+^ **(H)** CD4 T cells are shown in HVL and LVL patients. *P*-values of Mann–Whitney tests are indicated on each panel.

Two groups of patients were next segregated based on the level of plasma viral load at BL: (i) a group with a high viral load (“HVL patients,” *n* = 13), i.e., plasma HIV-RNA levels > median (5.65 log copies/mL) and (ii) a group with a low viral load (“LVL patients,” *n* = 14), i.e., plasma HIV-RNA levels ≤ median (Figure 2A). HVL patients exhibited higher CD8 T-cell activation (*p* = 0.024, Figure 2B) and showed lower peripheral CD4 T-cell counts (*p* = 0.0002, Figure 2C) as compared with LVL patients. HVL patients also exhibited higher CD4 T-cell activation (Figure 2D). Of note, although T-cell activation (% HLA-DR^+^CD38^+^ in CD8 T cells) and plasma viral load were strongly correlated in this cohort of patients [*R* = 0.66 and *p* = 0.0002 (16)], HIA and HVL groups were not identical as 5/14 (36%) patients from the HIA group belonged to the LVL group, suggesting that immune activation and viral load are correlated but independent variables (Fisher’s exact test: *p* = 0.12).

**Figure 3:**
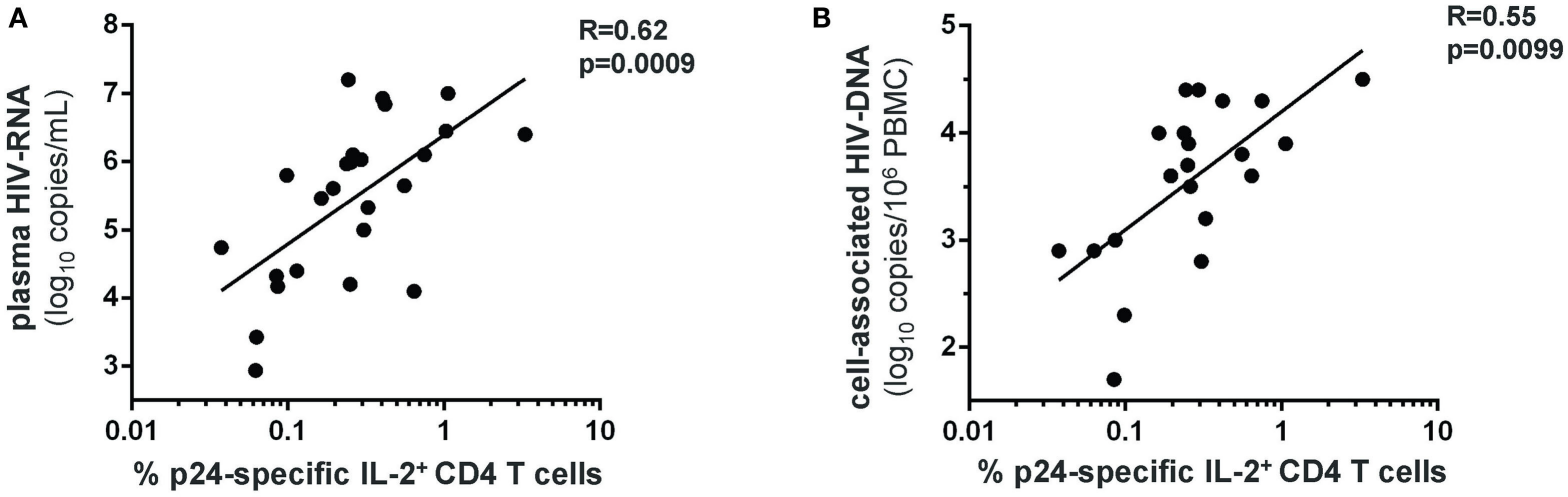
**Levels of HIV-specific IL-2^+^ CD4 T cells strongly correlate with the plasma viral load and the blood HIV reservoir size in early PHI**. Frequencies of gag-p24-specific IL-2^+^ CD4 T cells in patients with PHI (at baseline) were correlated to plasma HIV-RNA levels **(A)**, and to total cell-associated HIV-DNA levels in the blood **(B)**. Spearman’s rank correlation coefficients (*R*) and corresponding *p*-values are indicated on each panel.

HIV-specific CD4 T-cell responses were measured following 72 h stimulation with the HIV-1 gag p24 protein (Figure S1B in Supplementary Material). HVL patients showed significantly higher frequency of IFN-γ^+^, IL-2^+^, IL-17^+^, and IFN-γ^+^IL-17^+^ HIV-specific CD4 T cells compared with LVL patients (Figures 2E–H). Moreover, we found a direct relationship between the frequency of IL-2-producing HIV-specific CD4 T cells and both the plasma viral load (Figure 3A) and the size of blood HIV reservoirs as assessed by total cell-associated HIV-DNA (Figure 3B).

Of note, following PMA/ionomycin stimulation, there was no difference in Th1 responses between both groups. There was a trend to a lower frequency of IL-17-expressing CD4 T-cells in HVL patients compared with LVL patients (data not shown).

### Effect of Early ART Initiation on HIV-Specific CD4 T-Cell Responses

We next assessed HIV-specific responses at month 6 of follow-up and compared untreated patients and ART-treated patients. At month 6, all treated patients had plasma HIV-RNA levels <400 copies/mL.

**Figure 4:**
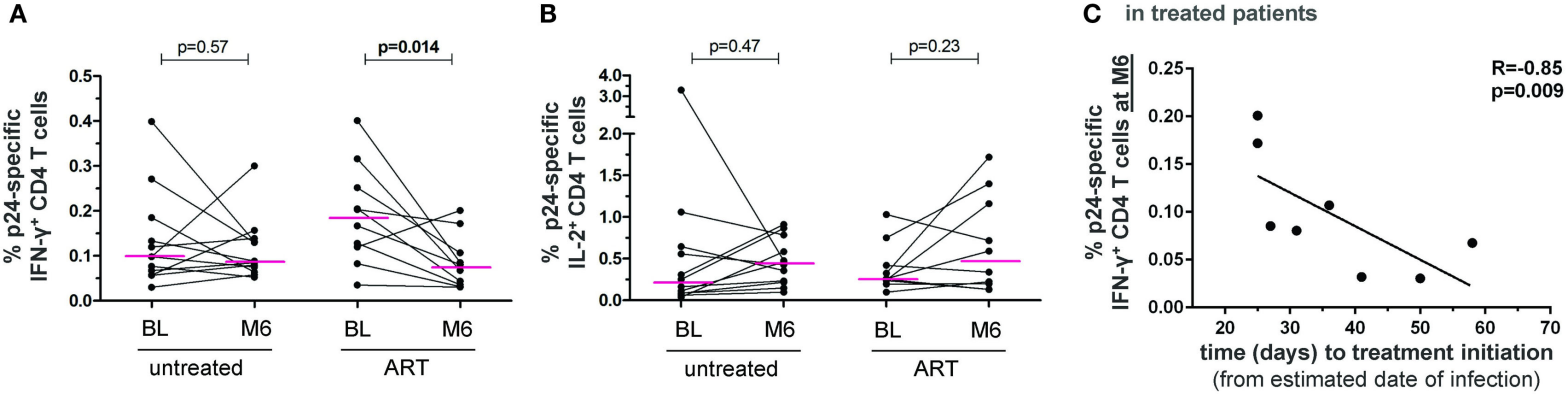
**HIV-specific CD4 T-cell responses follow-up in untreated and ART-treated patient with PHI**. Frequencies of HIV-1 gag-p24-specific IFN-γ^+^ **(A)** and IL-2^+^ **(B)** CD4 T cells in patients at baseline (BL) and month 6 of follow-up (M6) in both untreated patients and patients receiving ART after enrollment. Wilcoxon rank tests were performed and *p*-values are indicated between time points. **(C)** The frequency of HIV-1 gag-p24-specific IFN-γ^+^ CD4 T cells at month 6 of follow-up in treated patients was correlated to the time interval between estimated date of infection and treatment initiation. The Spearman’s rank correlation coefficient (*R*) and corresponding *p*-value are indicated.

Interestingly, the frequency of IFN-γ^+^ − but not of IL-2^+^ − p24-specific CD4 T cells significantly decreased between BL and month 6 in ART-treated patients but not in untreated patients (Figures 4A,B). There was also a trend to a decrease in IL-17^+^ p24-specific CD4 T cells only in ART-treated patients (*p* = 0.08), which was significant when considering CCR6^+^IL-17^+^ cells (*p* = 0.019) (data not shown).

In addition, we evaluated the impact of the time interval between estimated date of infection and treatment initiation. Notably, the level of IFN-γ^+^ p24-specific CD4 T cells at month 6 negatively correlated with the time to treatment (Figure 4C). Thus, although the ART-mediated control of viral burden reduced the level of HIV-specific CD4 T cell IFN-γ responses, early treatment initiation may allow patients to better maintain these responses.

## Discussion

This study highlights interrelationships between immune activation, viral replication and T-cell responses in PHI. We showed that a high immune activation level in PHI was associated with lower polyclonal IFN-γ^+^ and IL-17^+^ T-cell responses, while no association was found with HIV-specific T-cell frequencies. Conversely, we found that a high viral load was associated with higher HIV-specific Th1 and Th17 cell responses. Besides, ART-mediated viral control was associated with a decrease in IFN-γ-producing HIV-specific CD4 T cells from BL to M6, although the earlier ART was introduced, the higher was the residual IFN-γ^+^ HIV-specific CD4 responses. These data highlight that early viral replication induces HIV-specific CD4 T cells, but also systemic immune activation associated with a down modulation of the functional capacities of bulk CD4 T-cells.

Generalized immune activation is known to be a major contributor to HIV-1 pathogenesis (20). The potential causes of immune activation in chronic HIV infection are well described and include a direct effect of the virus or mediated by HIV viral proteins, an homeostatic response to CD4 lymphopenia, innate and adaptive immune responses, microbial translocation and reactivation of other viruses (e.g., CMV) [reviewed in Appay and Sauce (21)]. The mechanisms leading to immune activation in PHI have been less clearly deciphered. We show here that levels of HIV-specific responses are not associated with peripheral CD8 T-cell activation. We previously demonstrated that microbial translocation does not occur at the time of PHI (16). We also reported the strong relationship between innate responses and CD8 T-cell activation (16). We may rule out a role for CD4 T-cell proliferation as a result of CD4 lymphopenia. Indeed, both groups HIA and LIA exhibited similar CD4 cell counts and did not significantly differ regarding CD4 T-cell proliferation as measured by Ki-67 expression. Altogether, our data support the hypothesis that, in PHI, systemic T-cell activation is primarily driven by HIV itself and the consequent innate immune activation rather than microbial translocation or CD4 T-cell responses.

We found that patients with HIA exhibited higher levels of CD4 T-cell activation and lower peripheral polyclonal effector Th1 and Th17 CD4 T-cell responses. Low polyclonal effector CD4 T-cell responses could result from early functional impairment of bulk memory CD4 T cells. Alternatively, it could be the consequence of a sequestration of memory CD4 T lymphocytes in lymph nodes and/or gastrointestinal tract, preferential sites of viral replication.

While HIV-specific effector CD4 T-cell responses were similar between HIA and LIA patients, higher HIV-specific Th1, Th17, and Th1/Th17 responses were detected among HVL patients compared to LVL patients. This underlines that these cells are driven by the viral burden. Accordingly, IFN-γ-specific CD4 T cells significantly decreased following introduction of antiretroviral treatment. In contrast, specific IL-2^+^ CD4 cells remained unchanged or increased in few patients, although they correlated with viral load at BL. This is in line with data reported by Harari et al. in chronic HIV infection indicating that prolonged ART diminished IFNγ- but increased IL-2-producing Gag-specific CD4 T cells (22). We may speculate that IFN-γ^+^ cells are part of the acute inflammatory response to the virus, while IL-2 is more long-term expressed, even after viral suppression, alongside specific CD4 T-cell proliferation, which is also rescued under ART (3). In untreated patients, the frequency of IFN-γ^+^ and IL-2^+^ cells remained unchanged between BL and month 6 of follow-up for most patients, indicating that patients were assessed at a time close to the peak of CD4 cell response, in accordance with a previous report (11).

While proliferative and IFN-γ^+^ HIV-specific CD4 responses were extensively described, the presence of HIV-specific Th17 cells has been debated. Indeed, Th17 cells are involved in the defense against bacteria and fungi. Virus-specific Th17 cells were not detectable in patients with chronic untreated HIV infection (23). Yue et al. showed that HIV-specific IL-17-producing CD4 T cells were low/undetectable in chronic HIV infection but detectable in early infection suggesting that these cells are depleted during chronic infection (24). As CMV-specific Th17 cells were also found in early HIV infection, but not in the chronic phase (where CMV-specific Th1 cells are although still detectable), there might be an inflammatory milieu due to high viral replication in acute infection, that may aberrantly prime virus-specific T cells to produce IL-17 (24). Of note, most IL-17^+^ CD4 T cells expressed CCR6 (Figure S1C in Supplementary Material) and were found to decrease with viral suppression under ART. This chemokine receptor is regulating Th17 cell migration to the gut-associated lymphoid tissue and identifies preferential HIV target cells (25).

The positive relationship that we and others (11) observed between the intensity of specific CD4 T-cell responses and plasma viral load suggests that CD4 responses do not contribute to the control of viral replication in this early phase of infection. This does not rule out, however, that early anti-HIV CD4 T cells are important for the subsequent establishment of a low viral set point (4) and have a beneficial influence on disease outcome, as shown in the SPARTAC trial (12). Accordingly, the crucial role of CD4 T cells has recently been highlighted by the identification of HLA class II alleles associated with high or low viremia (5), and by the delineation of immune correlates of protection in the RV144 vaccine trial (8). The beneficial outcome of CD4 T-cell help most likely relies on the ability of cytokine-secreting antigen-specific CD4 T-cells (i) to rescue exhausted CD8 T cells, as shown in a model of chronic viral infection (26), and (ii) to provide B-cell help to mount effective antibody responses, as emphasized by HIV vaccine studies (8, 9). In light of that, the negative correlation between time to ART initiation after infection and the levels of IFN-γ^+^ HIV-specific CD4 responses maintained at month 6 further supports the importance of early treatment.

Taken together, these data illustrate the complex interrelationships between virus, immune activation and CD4 T-cell responses. Of note, patients with high viral load who exhibited higher effector CD4 T-cell responses could even more benefit from the early introduction of ART, allowing better maintenance of protective HIV-specific CD4 T cell responses.

## Author Contributions

LW and MC conceptualized and designed the study; LW, MC, FB-S, and DS-A contributed to the experimental design and provided intellectual input; MC and CD performed experiments; PC, P-MG, and MM included patients and provided clinical data; MC and LW analyzed data and wrote the manuscript.

## Conflict of Interest Statement

The authors declare that the research was conducted in the absence of any commercial or financial relationships that could be construed as a potential conflict of interest.

## Supplementary Material

Figure S1 **Representative plots of intracellular cytokine staining (ICS)**. CD4^+^ T cells were isolated from PHI patients and stimulated with PMA and ionomycin for 5 h **(A)** or co-cultured with autologous monocytes and with or without recombinant HIV gag p24 protein **(B)**. ICS was performed to assess expression of the indicated cytokines in CD3^+^CD4^+^ cells, and boolean gates were used for the analysis. **(C)** CCR6 expression is shown on IL-17^+^ cells (open histogram) as compared to CD4 T cells (filled histogram) following PMA and ionomycin stimulation (left panel) or p24 specific stimulation (right panel). Most IL-17^+^ cells express CCR6 corroborating IL-17 staining specificity.
