# Supplementary figures and images for: CD4 T-Cell Responses in Primary HIV Infection: Interrelationship with Immune Activation and Virus Burden
Source: Front Immunol. 2016 Sep 29;7:395. doi: 10.3389/fimmu.2016.00395 (PMC5040706; doi:10.3389/fimmu.2016.00395)

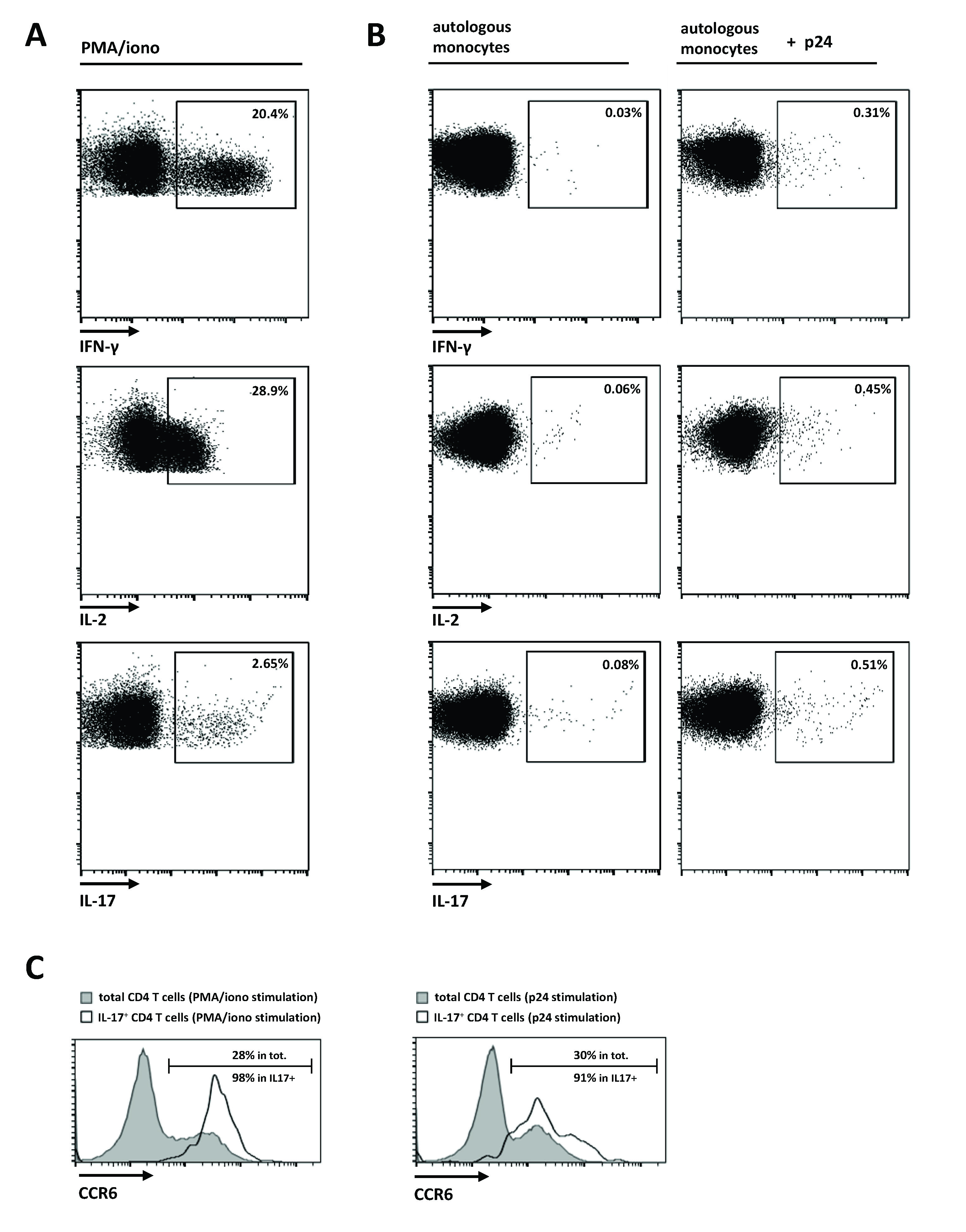

Supplement: Figure S1 — Representative plots of intracellular cytokine staining (ICS). CD4+ T cells were isolated from PHI patients and stimulated with PMA and ionomycin for 5 h (A) or co-cultured with autologous monocytes and with or without recombinant HIV gag p24 protein (B). ICS was performed to assess expression of the indicated cytokines in CD3+CD4+ cells, and boolean gates were used for the analysis. (C) CCR6 expression is shown on IL-17+ cells (open histogram) as compared to CD4 T cells (filled histogram) following PMA and ionomycin stimulation (left panel) or p24 specific stimulation (right panel). Most IL-17+ cells express CCR6 corroborating IL-17 staining specificity. [file image_1.jpg]
